# Prevalence of myocarditis and cardiotropic virus infection in Africans with HIV-associated cardiomyopathy, idiopathic dilated cardiomyopathy and heart transplant recipients: a pilot study

**DOI:** 10.5830/CVJA-2013-039

**Published:** 2013-08

**Authors:** Gasnat Shaboodien, Mpiko Ntsekhe, Patrick J Commerford, Motasim Badri, Bongani M Mayosi, Christopher Maske, Helen Wainwright, Heidi Smuts

**Affiliations:** The Cardiac Clinic and the Hatter Institute for Cardiovascular Research in Africa, Department of Medicine, Groote Schuur Hospital and University of Cape Town, Cape Town, South Africa; The Cardiac Clinic and the Hatter Institute for Cardiovascular Research in Africa, Department of Medicine, Groote Schuur Hospital and University of Cape Town, Cape Town, South Africa; The Cardiac Clinic and the Hatter Institute for Cardiovascular Research in Africa, Department of Medicine, Groote Schuur Hospital and University of Cape Town, Cape Town, South Africa; The Cardiac Clinic and the Hatter Institute for Cardiovascular Research in Africa, Department of Medicine, Groote Schuur Hospital and University of Cape Town, Cape Town, South Africa; College of Medicine, King Saud Bin Abdulaziz University for Health Sciences, Riyadh, Kingdom of Saudi Arabia; The Cardiac Clinic and the Hatter Institute for Cardiovascular Research in Africa, Department of Medicine, Groote Schuur Hospital and University of Cape Town, Cape Town, South Africa; Division of Anatomical Pathology, Department of Clinical Laboratory Sciences, National Health Laboratory Service and University of Cape Town, Cape Town, South Africa; Division of Anatomical Pathology, Department of Clinical Laboratory Sciences, National Health Laboratory Service and University of Cape Town, Cape Town, South Africa; Division of Medical Virology, Department Clinical Laboratory Sciences, National Health Laboratory Service and University of Cape Town, Cape Town, South Africa

**Keywords:** HIV-associated cardiomyopathy, myocarditis, dilated cardiomyopathy, cardiotropic virus

## Abstract

**Background:**

The prevalence of myocarditis and cardiotropic viral infection in human immunodeficiency virus (HIV)-associated cardiomyopathy is unknown in Africa.

**Methods:**

Between April 2002 and December 2007, we compared the prevalence of myocarditis and cardiotropic viral genomes in HIV-associated cardiomyopathy cases with HIV-negative idiopathic dilated cardiomyopathy patients (i.e. negative controls for immunodeficiency) and heart transplant recipients (i.e. positive controls for immunodeficiency) who were seen at Groote Schuur Hospital, Cape Town, South Africa. Myocarditis was sought on endomyocardial biopsy using the imunohistological criteria of the World Heart Federation in 33 patients, 14 of whom had HIV-associated cardiomyopathy, eight with idiopathic dilated cardiomyopathy and 11 heart transplant recipients.

**Results:**

Myocarditis was present in 44% of HIV-associated cardiomyopathy cases, 36% of heart transplant recipients, and 25% of participants with idiopathic dilated cardiomyopathy. While myocarditis was acute in 50% of HIV- and heart transplant-associated myocarditis, it was chronic in all those with idiopathic dilated cardiomyopathy. Cardiotropic viral infection was present in all HIV-associated cardiomyopathy and idiopathic dilated cardiomyopathy cases, and in 90% of heart transplant recipients. Multiple viruses were identified in the majority of cases, with HIV-associated cardiomyopathy, heart transplant recipients and idiopathic dilated cardiomyopathy patients having an average of 2.5, 2.2 and 1.1 viruses per individual, respectively.

**Conclusions:**

Acute myocarditis was present in 21% of cases of HIV-associated cardiomyopathy, compared to none of those with idiopathic dilated cardiomyopathy. Infection with multiple cardiotropic viruses may be ubiquitous in Africans, with a greater burden of infection in acquired immunodeficiency states.

## Abstract

Dilated cardiomyopathy is a common manifestation of HIV-associated cardiovascular disease in Africans.^1^ Little is known, however, about the cause of cardiomyopathy in HIV-infected people living in Africa, a continent with the largest number of people with HIV/AIDS in the world.^2^-^4^ A number of hypotheses regarding the pathogenesis of HIV-associated cardiomyopathy have been proposed including myocarditis due to direct infection with HIV or other cardiotropic viruses, genetic predisposition, and nutritional deficiencies.^4^-^6^

An autopsy study of 16 patients with HIV/AIDS from the Democratic Republic of the Congo reported histopathological changes of acute myocarditis in all cases, which were attributed to opportunistic infection with *Toxoplasma gondii* in three of 16 cases (18.75%), *Cryptococcus neoformans* in three of 16 cases (18.75%) and *Mycobacterium avium intracellulare* in two of 16 cases (12.5%), and to direct HIV infection in eight of 16 (50%) patients.^7^ This report raises the possibility that HIV-associated cardiomyopathy may be caused by potentially treatable opportunistic infections in up to 50% of patients living in the sub-Saharan region.^2^ By contrast, in Western series, opportunistic viral infections or idiopathic causes have been implicated in a significant proportion of cases of HIV-associated cardiomyopathy.^8^,^9^

To the best of our knowledge, there have been no ante-mortem studies on the prevalence of myocarditis and cardiotropic viral infection in patients with HIV-associated cardiomyopathy living in Africa.^2^-^4^ The aim of this study was to determine the prevalence and type of myocarditis and cardiotropic viral infection in patients with HIV-associated cardiomyopathy compared to those with idiopathic dilated cardiomyopathy and heart transplant recipients in Cape Town.

## Methods

This was a case-comparison study in which the frequency of myocarditis and cardiotropic viral infection in patients with HIV-associated cardiomyopathy was compared with those with idiopathic dilated cardiomyopathy without HIV infection (i.e. negative controls for acquired immunodeficiency state) and heart transplant recipients (i.e. positive controls for an acquired immunodeficiency state). The endomyocardial biopsy study was conducted in the cardiac catheterisation laboratory at Groote Schuur Hospital in Cape Town from April 2002 to December 2007, before anti-retroviral therapy became widely available in South Africa.

A minimum of four right ventricular endomyocardial biopsy specimens were obtained for all patients through the right internal jugular vein. The endomyocardial biopsy samples were immediately placed in formalin (for light microscopy) and snap frozen in liquid nitrogen (for the virological studies).

The study was approved by the Research Ethics Committee of the Faculty of Health Sciences of the University of Cape Town, and all participants gave written informed consent.

We recruited consecutive cases of HIV-positive and HIV-negative patients with a new-onset echocardiographically confirmed diagnosis of dilated cardiomyopathy (i.e. a left ventricular ejection fraction ≤ 45% and/or fractional shortening < 25%, plus a left ventricular end-diastolic diameter > 117% of the predicted value for age and body surface area by applying Henry’s formula)^10^ or isolated left ventricular systolic dysfunction (i.e. a left ventricular ejection fraction ≤ 45% and/or fractional shortening < 25% plus normal left ventricular dimensions).

Patients with secondary causes of cardiomyopathy or left ventricular systolic dysfunction (such as hypertension, valvular heart disease, diabetes, coronary artery disease, or prior AIDS defining opportunistic infection or malignancy) were excluded from the study. Coronary artery disease was excluded by coronary angiography in all cases with cardiomyopathy who participated in this study.

The HIV-positive patients with unexplained cardiomyopathy were categorised as HIV-associated cardiomyopathy, while the HIV-negative cases were labelled as idiopathic dilated cardiomyopathy. Heart transplant recipients who were undergoing routine endomyocardial biopsy to monitor rejection were also enrolled.

The diagnosis of myocarditis was based on the immunohistological criteria of the World Heart Federation.^11^,^12^ These criteria define an abnormal lymphocytic infiltrate as the presence of > 14 CD_3_^+^ lymphocytes per high-power field (and up to four CD_68_^+^ macrophages included in this total amount) (magnification, × 40) with use of a microscope with wide-field, 10 × eyepieces (diameter 550 m; area 2.4 × 105 m; Olympus BH-2, Hamburg, Germany).

Acute myocarditis was defined as a significant lymphocytic infiltrate with myocytolysis (i.e. necrosis or degeneration) on light microscopy, while chronic myocarditis was defined as a significant lymphocytic infiltration with no myocytolysis. Patients with no infiltrating cells or insignificant lymphocyte infiltrate (< 14 leukocytes/mm^2^) and no myocytolysis were classified as having no evidence of myocarditis.

Immunohistochemistry was used to define the types of immune cells in the inflammatory infiltrate of myocarditis. Formalin-fixed, paraffin-embedded tissues were sectioned at 5μm for routine, single-label immunohistochemistry. Cell populations were characterised using antibodies specific for CD_3_ (rabbit polyclonal, A 0452, DakoCytomation, Carpinteria, CA), CD_4_ (clone 1F6, VP-C318, Vector Laboratories, Burlingame, CA), CD_8_ (clone 1A5, VP-C325, Vector) and CD_68_ (clone KP1, M 0814, DakoCytomation).

Two methods were used for the detection of cardiotropic viruses: viral antigen detection using immunohistochemical methods and identification of viral genome by PCR or reverse transcription (RT)-PCR. To evaluate the extent to which cardiotropic viral infections may contribute to induction of the myocardial inflammatory response in the heart tissue under study, specific virological immunoperoxidase screening was conducted for seven cardiotropic viruses using the following virus-specific antigens: cytomegalovirus (Dako, Carpenteria, California, M0854), adenovirus (Novocastra, NCL-ADENO), herpes simplex virus types 1 and 2 (Dako, Carpenteria, California, B0114 and B0116), HIV-1 p24 antigen (Dako, Carpenteria, California, M0857), Epstein-Barr virus (Dako, Carpenteria, California, M0897), enterovirus (Dako Carpenteria, California, M7064), and parvovirus B19 (Dako Carpenteria, California, B0091), along with reviews of haematoxylin and eosin (H&E)- stained sections and special staining for histological evidence of infective agents, such as *Toxoplasma gondii*, *Cryptococcus neoformans* and mycobacteria.

Sections were deparaffinised in xylene and rehydrated through graded ethanols, followed by blocking of endogenous peroxidase by incubation in 3% hydrogen peroxide in phosphate-buffered saline. Antigen retrieval in most cases consisted of microwaving in citrate buffer. Antigen retrieval for CD_4_ consisted of microwaving in EDTA buffer (Lab Vision, Fremont, CA), for adenovirus it consisted of five-minute digestion with proteinase K (Dako, Carpenteria, California), and for CD_8_ it consisted of 20-min pressure cooker treatment in Trilogy solution (Cell Marque, Hot Springs, AK). Sections were incubated with primary antibody followed by an avidin–biotin block (Vector) to block endogenous biotin, and sequential incubation with biotinylated secondary antibody and horseradish peroxidase-conjugated avidin (ABC Standard or ABC Elite, Vector), or the EnVision polymer system (Dako, Carpenteria, California) applied according to the manufacturer’s instructions.

Antigen–antibody complex formation was detected by use of 3,3′- diaminobenzidine (DAB) chromogen (Dako, Carpenteria, California) and tissues were counterstained with Mayer’s haematoxylin. Viral antigen was detected with viral antibodies to cytomegalovirus, Epstein-Barr virus, herpes simplex virus, parvovirus B19, adenovirus, enterovirus and HIV-1 (Dako, Carpenteria, California; Novocastra, UK Laboratories) in the three groups. Control lymph node tissue was used as positive controls for each virus under study.

## Statistical analysis

Groups were compared using the Mann-Whitney non-parametric test, Kruskal-Wallis test, χ^2^ test or Fisher’s exact test, as appropriate. Statistical analysis of quantitative image analysis data was performed on pooled individual data points collected for each patient within the evaluated groups. All tests were two-sided and *p* < 0.05 was considered significant. Statistical analysis was performed using Statistica (version 7) and EpiInfo software.

## Results

Control endomyocardial biopsy samples were obtained from HIV-negative individuals who were undergoing routine endomyocardial biopsy in the Cardiac Catheterisation Laboratory at Groote Schuur Hospital, either for the investigation of idiopathic dilated cardiomyopathy or for monitoring of rejection in heart transplant recipients. The heart transplant recipients served as positive controls for immunosuppression (which is also present in HIV-associated cardiomyopathy) and their likelihood of having a high frequency of myocarditis resulting from varying degrees of rejection, whereas the idiopathic dilated cardiomyopathy cohort acted as negative controls for absence of immunosuppression and a likely low prevalence of active myocarditis.

During the study period, a total of 40 participants (15 with HIV-associated cardiomyopathy, 10 with idiopathic dilated cardiomyopathy and 15 heart transplant recipients) met the inclusion criteria and were subjected to the endomyocardial biopsy study. Of these, seven were excluded due to inadequate amounts of endomyocardial biopsy tissue for the histological, immunohistochemical and virological analysis. The clinical characteristics of participants who were analysed are presented in Table 1. There was no significant difference in age and gender between the cases with HIV-associated cardiomyopathy, idiopathic dilated cardiomyopathy and heart transplant recipients.

**Table 1:** Clinical Characteristic Of Patients With HIV-Associated Cardiomyopathy (HIVAC), Idiopathic Dilated Cardiomyopathy (IDCM) And Heart Transplant Recipients (HTX) Who Were Studied With Endomyocardial Biopsy

	*HIVAC (n = 14)*	*iDCM (n = 8)*	*HTx (n = 11)*	p*-value^‡^*
Median age in years (range)	40 (27–63)	43.5 (31–54)	50 (26–69)	NS*
Male (%)	58	75	82	0.44
Median CD_4_^+^ T-cell count (cells/mm^3^)	253 (200–344)	not applicable	not applicable	
Median HIV load, (copies/ml)	155 000 (28 000-265 000)	not applicable	not applicable	
Median LVEF (%)	30.6 (23–35)	27 (13.5–29)	56.5 (46.8–59.0)	< 0.0001
Median duration of illness (weeks)	18 (14.5–26)	14 (7–18)	18.5 (6–21.8)	0.3

^‡^*p*-value: Fisher’s exact test for categorical variables, Kruskal-Wallis test for continuous variables. LVEF, left ventricular ejection fraction, NS, not significant.

Fifteen patients with HIV-associated cardiomyopathy underwent endomyocardial biopsy, but one patient with inadequate biopsy material was excluded from the analysis, resulting in 14 patients being entered into the analysis. All cases were of black African descent (mean age, 40.5 ± 10.7). A low CD_4_^+^ T-cell count (mean cell count 246 ± 193 cells/mm^3^; normal range, 600–1000 cells/mm^3^) and left ventricular ejection fraction (mean range 27.9 ± 6.92%) were found in the patients with HIV-associated cardiomyopathy, as expected.^6^

Ten consecutive patients with idiopathic dilated cardiomyopathy were recruited and used for comparison with findings in the patients with HIV-associated cardiomyopathy. Two patients were excluded from the analysis due to inadequacy of endomyocardial biopsies, resulting in eight patients being entered in the comparative analysis (mean age 43 ± 7.35 years; mean left ventricular ejection fraction 26.2 ± 10.39%).

Fifteen consecutive heart transplant recipients who were undergoing routine endomyocardial biopsy were used as controls for comparison with findings in the patients with HIV-associated cardiomyopathy. Due to tissue inadequacy, four patients were excluded from the analysis, resulting in 11 patients being entered into the analysis (mean age 47.1 ± 14.1 years; mean left ventricular ejection fraction 52.8 ± 7.7%).

**Fig. 1.**
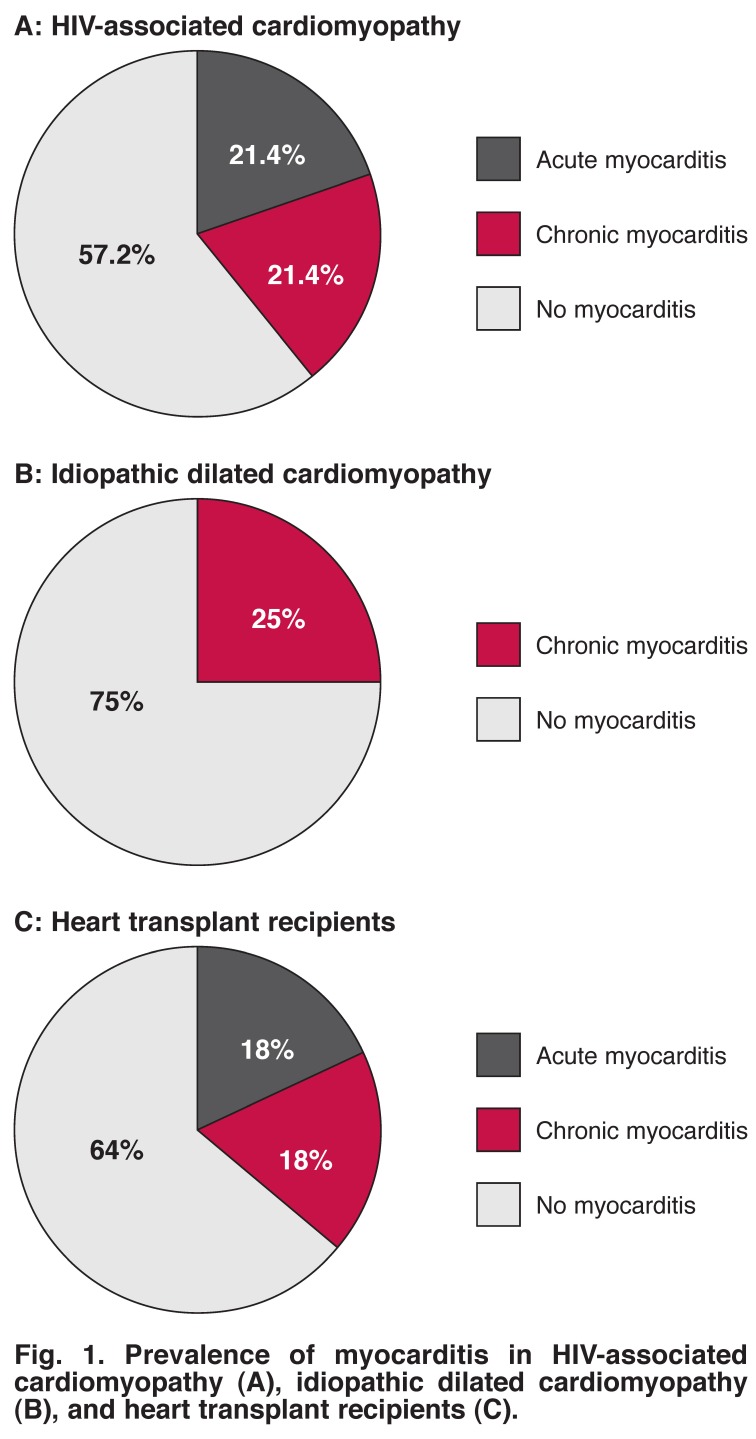
Prevalence of myocarditis in HIV-associated cardiomyopathy (A), idiopathic dilated cardiomyopathy (B), and heart transplant recipients (C).

Immunohistochemical analysis of the mononuclear infiltrates in the myocardium demonstrated a lymphocyte population expressing the pan T-cell marker CD_3_, CD_4_^+^ T lymphocytes (helper/inducer), CD_8_^+^ T lymphocytes (cytotoxic/suppressor), and monocytes and macrophages (CD_68_^+^ cells). Using CD_3_ and CD_68_ immune cell counts, we determined that six of 14 (42.8%) of the HIV-associated cardiomyopathy cohort met the World Heart Federation criteria for myocarditis, displaying > 14 leukocytes/mm^2^; three cases of 14 had acute myocarditis (21.4%), while three of 14 had chronic myocarditis (21.4%) (Fig. 1A). The remaining eight cases did not display any myocarditis.

Two of the eight idiopathic dilated cardiomyopathy cases (25%) were classified as having chronic myocarditis; the other six samples were classified as having no myocarditis (Fig. 1B). None of the idiopathic dilated cardiomyopathy cases met the criteria for acute myocarditis.

Four heart transplant recipients met the criteria for myocarditis: two of 11 cases (18%) had acute myocarditis and two of 11 (18%) displayed chronic myocarditis (Fig. 1C). There was no significant difference in the prevalence of chronic myocarditis between the groups (*p* = 0.48).

**Fig. 2.**
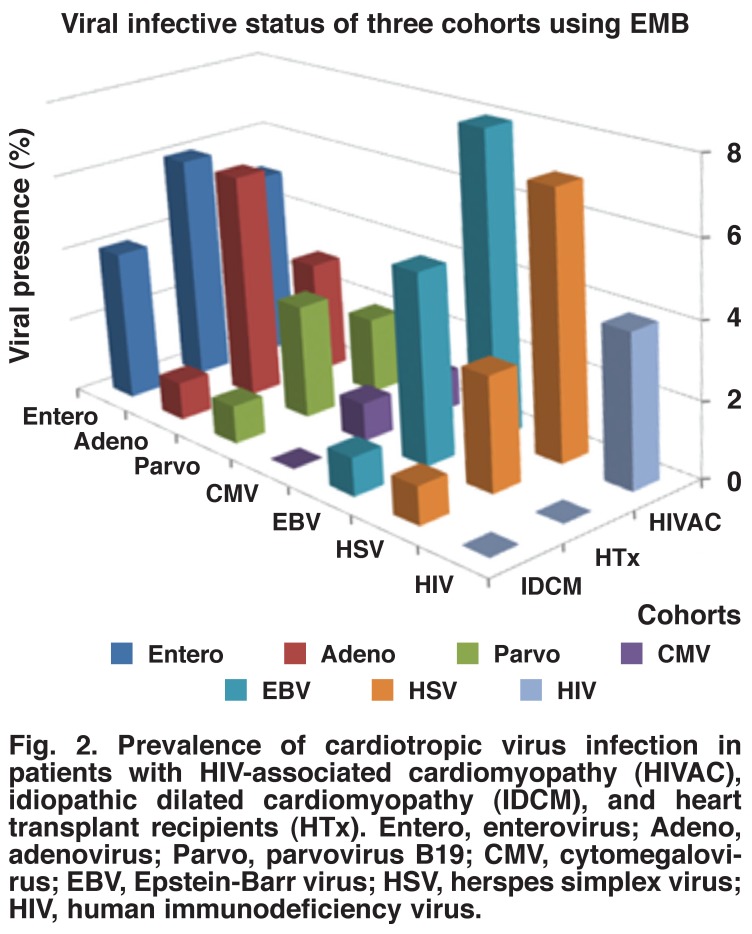
Prevalence of cardiotropic virus infection in patients with HIV-associated cardiomyopathy (HIVAC), idiopathic dilated cardiomyopathy (IDCM), and heart transplant recipients (HTx). Entero, enterovirus; Adeno, adenovirus; Parvo, parvovirus B19; CMV, cytomegalovirus; EBV, Epstein-Barr virus; HSV, herspes simplex virus; HIV, human immunodeficiency virus.

All cases of HIV-associated cardiomyopathy were infected with at least one cardiotropic virus based on results of testing for viral genomes by PCR. No virus was detected by immunohistochemical methods, suggesting low levels of viral particles in the endomyocardial biopsies. The number of viruses per case ranged from one to four with a mean viral burden of 2.5 viruses per case irrespective of whether patients were classified as having myocarditis or no myocarditis. The biopsy tissues from patients with HIV-associated cardiomyopathy were dominated by the Epstein-Barr virus (64%) and herpes simplex virus (50%) with parvovirus B19 (14%) and cytomegalovirus (7%) having the lowest frequency. HIV-1 was detected in 29% of the HIV-associated cardiomyopathy cases (Fig. 2).

Similarly, all cases of idiopathic dilated cardiomyopathy were infected with at least one cardiotropic virus, with a mean viral burden of 1.1 viruses per case. Enterovirus was the commonest virus (56%), followed by Epstein-Barr virus (29%). Herpes simplex virus, adenovirus and parvovirus B19 virus were each detected in 12% of the cases (Fig. 2). Cytomegalovirus infection was not detected in participants with idiopathic dilated cardiomyopathy.

A viral prevalence of 90% was found in the heart transplant recipients as 10 of the 11 cases were positive for at least one virus as detected by PCR. A mean viral burden of 2.2 viruses per case was found irrespective of whether patients were classified as having myocarditis or no myocarditis. The most prevalent viruses was the enterovirus (50%) and adenovirus (50%), followed by Epstein-Barr virus (32%) and herpes simplex virus (25%), with parvovirus B19 (18%) and cytomegalovirus (18%) being the least common agents (Fig. 2).

## Discussion

In this first report of the prevalence of myocarditis and cardiotropic viral infection in African patients with HIV-associated cardiomyopathy, we show that nearly half of the patients may have had acute and chronic myocarditis. By contrast, idiopathic dilated cardiomyopathy was associated with myocarditis in a quarter of the cases, none of whom had acute disease.

The presence of genomes of cardiotropic viruses was almost universal in African patients with HIV-associated cardiomyopathy, idiopathic dilated cardiomyopathy, and heart transplant recipients. Furthermore, we observed that participants who were immunosuppressed by HIV infection or on immunosuppressive treatment for heart transplantation had double the number of cardiotropic viruses per case, compared to those with idiopathic dilated cardiomyopathy (2.2–2.5 viruses per case compared to 1.1 virus per case).

Because heart biopsy and viral genome analysis are rarely done in many regions of the world, the prevalence of viral myocarditis in much of Africa, Asia, the Middle East and South America is unknown.^12^ The few endomyocardial biopsy studies in African patients with cardiomyopathy were conducted in the pre-HIV era.^13^–^16^ These studies, which did not use modern techniques of immunohistochemistry and molecular characterisation of viral genomes, had conflicting findings on the prevalence of myocarditis.

In the largest endomyocardial biopsy study of 76 South African patients with idiopathic dilated cardiomyopathy, no evidence of myocarditis was found, leading to the conclusion that the cardiac failure of dilated cardiomyopathy was due to an unknown functional abnormality, such as a toxin or metabolic defect.^13^,^14^ However, endomyocardial biopsy studies of dilated and peripartum cardiomyopathy from Kenya revealed that about half of the patients had evidence of healed myocarditis but no serological evidence of a previous Coxsackie virus infection or any other common viral infections.^15^,^16^ The authors concluded that the myocarditis was due to an inappropriate immunological reaction to myocardial muscle.^15^,^16^

The literature from developed countries on the presence of viral genomes in cardiomyopathy has pointed to enterovirus, adenovirus, cytomegalovirus and HIV-1 as the dominant opportunistic viral infections in the heart tissue of patients with HIV-associated cardiomyopathy.^17^–^19^ By contrast, our study revealed Epstein-Barr and herpes simplex viruses to be the most prevalent cardiotropic viruses in patients with HIV-associated cardiomyopathy.

Epstein-Barr virus and herpes simplex virus are common herpes viruses that have a latency stage; they are reactivated in immunocompromised individuals, causing tissue damage. Similarly, enteroviruses, which were common in our cases with HIV-associated cardiomyopathy and idiopathic dilated cardiomyopathy, are also known have a latency stage, which could have important consequences for the clinical course of the disease. Parvovirus B19 had the lowest prevalence in all groups of patients, whereas it is one of the commonest causes of viral myocarditis in North America and Europe.^20^,^21^ We found no obvious pattern of association with any of these viral genomes with myocarditis, in line with previous observations.^22^

While the phenomenon of multiple viral infections is known to occur in patients with end-stage dilated cardiomyopathy, the prevalence of cardiotropic viral infection is much higher in African patients (100%) compared to elsewhere in the world (~ 65%).^22^ It is of interest that the number of viruses per case ranged from one to four, with a mean viral burden of 2.3 types of viruses in HIV-associated cardiomyopathy, zero to four in heart transplant recipients with a mean viral burden of 2.2 types per case, and one to two in those with idiopathic dilated cardiomyopathy with a mean viral burden of 1.1 per case. These findings, which suggest an increased susceptibility to viral infection in immunosuppressed groups, point to the possibility that the damage to the myocardium in patients with cardiomyopathy is not only through post-viral autoimmune mechanisms but may also be as a result of repeated infections by different cardiotropic viruses.

The finding of myocarditis in a significant proportion of patients with idiopathic dilated cardiomyopathy may have prognostic implications. Kindermann and colleagues showed that the risk of death or need for cardiac transplantation in patients with myocarditis was worse in those with inflammation than in those without, as assessed by immunohistology.^23^ Significant inflammation was present in 25% of the participants who underwent endomyocardial biopsy testing as part of investigation of unexplained dilated cardiomyopathy.

It is essential to determine whether the cellular and imunohistological changes have prognostic effects in African patients with dilated cardiomyopathy. Furthermore, interferon-beta treatment may eliminate enterovirus and adenovirus and improve left ventricular function in immuno-competent patients with myocardial persistence of viral genomes and left ventricular dysfunction.^20^ There is a need to assess the effectiveness of interferon-beta treatment in African patients with cardiomyopathy, in the light of the discovery of the overwhelming presence of viral genomes in our patients.

The major weakness of the study was the small sample size. The number of analysed patients may have beeen insufficient to reliably determine the proportion of patients with HIV-associated cardiomyopathy with acute myocarditis, or to delineate differences between the HIV-infected and HIV-negative patients with dilated cardiomyopathy. However, while the small sample size limits the generalisability of the findings, it provides a strong basis for the exploration of the findings in larger studies.

Furthermore, the use of a prospective consecutive design with two different types of controls has, however, ensured that the study has internal validity with regard to the diagnosis of myocarditis and patterns of viral infection in immunosuppressed hosts. The heart transplant recipients, as expected, had evidence of acute myocarditis due to varying degrees of rejection. It was also interesting to note that the burden of viral infection was higher in the immunosuppressed patients (by HIV and heart transplantation), with cytomegalovirus infection being observed, as expected, only in immunosuppressed individuals.^24^ We included all eligible patients with HIV-associated cardiomyopathy during the period of the study (five years), and the sample size was not different from that of similar studies.^25^

The prevalence of myocarditis in HIV-associated cardiomyopathy in our study is similar to the rates found in North America in the pre-HIV era, and the frequency of chronic myocarditis in idiopathic dilated cardiomyopathy is consistent with findings elsewhere in the world.^8^,^26^ The external validity of this study is confirmed by the finding of a high prevalence of enteroviruses in HIV-positive heart tissue, which corresponds with data that were reported by Herskowitz in patients with AIDS nearly two decades ago.^8^

## Conclusion

Myocarditis and cardiotropic viral infection appear to be common and may play a significant role in the pathogenesis of HIV-associated and idiopathic dilated cardiomyopathy in Africans. The results of this small pilot study need to be confirmed in larger studies to endorse these observations and determine the prognostic and therapeutic implications of our findings. If the pathogenetic role of cardiotropic viral infection in cardiomyopathy is confirmed in other studies, this might lead to the development of appropriate immunisation to aid the prevention of the disease and reduce the prevalence of cardiomyopathies, which are endemic in Africa.^27^
